# Utilization of inpatient medical care in Germany

**DOI:** 10.17886/RKI-GBE-2017-128

**Published:** 2017-12-13

**Authors:** Franziska Prütz, Alexander Rommel

**Affiliations:** Robert Koch Institute, Department of Epidemiology and Health Monitoring, Berlin

**Keywords:** HOSPITAL, ADULTS, HEALTH SERVICES RESEARCH, HEALTH MONITORING, GERMANY

## Abstract

Inpatient care is an important pillar of the health care system. Data from health surveys enable to analyse the utilization of inpatient treatment from the patient’s perspective, and to identify associations to social determinants and other influencing factors. As part of the GEDA 2014/2015-EHIS study, data was collected for the indicator ‘utilization of inpatient medical care during the last 12 months’. The data analysis shows that 16.9% of women and 15.3% of men were admitted to hospital at least once during the last 12 months. The utilization of inpatient treatment depends on age: among people aged 65 or above, 25.9% of women and 25.8% of men were admitted to hospital during the last 12 months. Almost no significant differences in utilization were identified in regard to gender, with the exception of the 18-to-29 age group, where admittance to hospital was more common among women than men. In terms of education, people with the lowest level of education have a higher utilization of inpatient care, and this is particularly the case in middle age.

## Introduction

Inpatient care constitutes an important pillar of the health care system. Alongside providing treatment to patients, hospitals are also tasked with undertaking clinical research, training health care professionals and teaching. Over a quarter of the total health expenditures is spent on hospital treatment. In 2015, this amounted to EUR 89.5 billion [1]. Hospital statistics registered around 19.8 million cases of people being hospitalised in 2015 [2].

Data on inpatient treatment can be analysed using data from official statistics (hospital statistics, Diagnosis Related Groups (DRG) statistics). However, it is only possible to identify the total number of hospitalisations that occurred from these data: if the same person is treated several times in a hospital within the same year, each visit is recorded in the statistics separately. In contrast, accounting data gained from health insurers can be used to study individual utilization; however, these data are often only available to a limited extent. Data from health surveys enable utilization to be mapped on an individual basis, regardless of a person’s insurance status, and can be used to analyse the association between inpatient treatment and social and other influencing factors. Furthermore, health surveys also collect data on services that are not covered by health insurance and thus are not included in official data sources [3-5].

Current data on the self-reported use of inpatient treatment was collected for the German Health Update (GEDA 2014/2015-EHIS) study. The study is part of the Health Monitoring framework at the Robert Koch Institute (RKI) as well as the system of European health monitoring; the questions asked for the European Health Interview Survey (EHIS) were integrated into the GEDA study.

**GEDA 2014/2015-EHIS**

**Data holder:** Robert Koch Institute

**Aims:** To provide reliable information about the population’s health status, health-related behaviour and health care in Germany, with the possibility of a European comparison

**Method:** Questionnaires completed on paper or online

**Population:** People aged 18 years and above with permanent residency in Germany

**Sampling:** Registry office sample; randomly selected individuals from 301 communities in Germany were invited to participate

**Participants:** 24,016 people (13,144 women; 10,872 men)

**Response rate:** 26.9%

**Study period:** November 2014 - July 2015

**Data protection:** This study was undertaken in strict accordance with the data protection regulations set out in the German Federal Data Protection Act and was approved by the German Federal Commissioner for Data Protection and Freedom of Information. Participation in the study was voluntary. The participants were fully informed about the study’s aims and content, and about data protection. All participants provided written informed consent.

More information in German is available at www.geda-studie.de

## Indicator

In the GEDA 2014/2015-EHIS study, data for the indicator ‘utilization of inpatient medical treatment during the last 12 months’ was collected using a self-reporting questionnaire that the participants completed on paper or online. It included the following question: ‘Have you been in hospital as an inpatient, that is overnight or longer, during the last 12 months? Do not include treatment in an accident and emergency ward or outpatient treatment in hospital.’

The following analyses are based on data from 23,901 people aged 18 or above (13,076 women; 10,825 men) with valid data on the utilization of hospital treatment in the last 12 months. The calculations were carried out with a weighting factor that corrected the sample for deviations from the German population structure (as of 31 December 2014) in terms of gender, age, district type and level of education. The district type reflects the degree of urbanisation and corresponds to the regional distribution in Germany. The International Standard Classification of Education (ISCED) was used to classify educational and occupational qualifications [6]. Differences between groups are interpreted as statistically significant when the respective confidence intervals do not overlap.

A detailed description of the methodology used for GEDA 2014/2015-EHIS can be found in Lange et al. 2017 [7] as well as in the article German Health Update: New data for Germany and Europe in issue 1/2017 of the Journal of Health Monitoring.

## Results and discussion

Approximately one sixth of the adult population (16.9% of women and 15.3% of men) were admitted to hospital for at least one night during the 12 months that preceded the study (Table 1). However, the use of hospital treatment is strongly dependent on age. Among people aged 65 or above, about a quarter of adults (25.9% of women and 25.8% of men) were admitted to hospital during the 12 months that preceded the study, which is almost twice as many as the rate identified among 45- to 64-year-olds.

There are almost no significant differences between women and men with regard to the utilization of hospital treatment. The only relevant difference occurs in the 18-to-29 age group, where significantly more women (15.1%) than men (8.7%) reported that they had been admitted to hospital in the 12 months preceding the study. The higher utilization of hospital treatment among women can be attributed to pregnancy and childbirth; this is clear from the hospital statistics held by the Federal Statistical Office [8]. This fact also explains the lower utilization of hospital treatment among women with higher levels of education, since university graduates are more likely to remain childless or to start a family at an older age [9].

Differences according to educational level are particularly evident among the 30-to-44 and 45-to-64 age groups. The proportion of people who were admitted to hospital at least once during the 12 months that preceded the study was higher in the lower educational group than in the group with the highest level of education (Table 1). This can be explained by the fact that many chronic diseases, such as cardiovascular diseases, mental disorders or musculoskeletal diseases, are more common among people with lower levels of education [10], which, in turn, leads to more hospitalisations. Significant differences in hospitalisation rates according to education were also identified among women aged 18 to 29, with 22.0% in the lower educational group, 15.9% in the medium educational group, and 7% in the highest educational group having been admitted to hospital in the 12 months preceding the study.

Hardly any significant differences in utilization were identified between the federal states. However, the level of hospitalisation among women living in Baden-Württemberg during the 12 months preceding the study is significantly lower than the national average. The same applies to men in Bremen. The data on the regional distribution of the utilization of inpatient services according to federal states are available from the Information System of the Federal Health Reporting (www.gbe-bund.de).

The proportion of adults who reported having been admitted to hospital in the 12 months preceding the study was almost the same as the figure gained by the GEDA survey conducted in 2012 (15.9% of women, and 15.8% of men) [11]. Between GEDA 2012 and GEDA 2014/2015-EHIS, the survey mode changed from a telephone to a paper or online survey. However, the survey method does not significantly affect indicators covering the utilization of health services [12].

There are, of course, a number of factors that may lead to errors or the underreporting of hospitalisations in health surveys. For example, very old or severely ill people are often unable to participate in surveys, and since these people use hospital services frequently, the figures described here need to be treated with caution. Recall bias can also constitute a source of error. However, hospitalisations are major events and are therefore usually remembered well. Recall bias is also more likely to occur when data is collected on periods that stretch to longer than 12 months [13]. A further limitation is caused by the fact that the reasons behind admittance to hospital are not recorded in the GEDA 2014/2015-EHIS data, making diagnosis or indication-specific analyses impossible.

A comparison between the results from GEDA 2014/2015-EHIS with those from official statistics is only possible to a limited extent. Developments in the number of cases are only partially reflected by hospital statistics because these do not indicate whether the same person was admitted to hospital on several occasions. Hospital statistics demonstrate that there were 23 cases per 100 inhabitants who were in hospital at least one day in 2015 [2]. This figure also includes under-18s, who are not included in the GEDA survey. Nevertheless, there is a certain degree of similarity between the results of the two studies when age groups are compared: in the age group between 15 and 24 years 15 women and 9 men per 100 inhabitants were admitted to hospital. Large differences are seen among the elderly: in the age group 65 or above 46 women and 53 men per 100 inhabitants were admitted to hospital. The main reasons behind this difference are the fact that elderly people are more likely to be admitted to hospital more than once in the same year and the possibility that surveys may underreport hospitalisations.

The indicator ‘utilization of inpatient medical treatment during the last 12 months’ maps the utilization of inpatient medical services and its links to socio-demographic factors. As such, it complements the indicators gained from official statistics and accounting data for issues such as hospital planning. Hospital planning is also affected by other factors including demographic change, medical and technological progress, changes in the disease spectrum, as well as shifts from inpatient care to the outpatient sector [14].

Together with the other contributions to this issue (Fact sheets on the utilization of outpatient medical care, the utilization of physiotherapy, the utilization of medically prescribed medicines and self-medication, and the Focus on the use of psychotherapeutic and psychiatric treatment) this Fact sheet provides an overview of essential aspects related to the utilization of health care by adults in Germany.

## Key statements

- 17% of women and 15% of men were admitted to hospital at least once during the past 12 months.
- 15% of women and 9% of men aged between 18 and 29 were admitted to hospital during the last 12 months; around a quarter of people aged 65 or above were hospitalised during this period.
- The proportion of women aged between 18 and 29 who needed inpatient treatment is significantly higher than in men due to pregnancy and childbirth.
- The proportion of middle aged adults who were admitted to hospital during the last 12 months is higher among people with lower levels of education than among the group with the highest level of education.

## Figures and Tables

**Table 1:** 12-month prevalence of utilization of hospital treatment according to gender, age and educational level (n=13,076 women; n=10,825 men) Source: GEDA 2014/2015-EHIS

Women	%	(95% CI)	Men	%	(95% CI)
**Women (total)**	**16.9**	**(16.1-17.8)**	**Men (total)**	**15.3**	**(14.5-16.2)**
**18-29 Years**	15.1	(13.1-17.3)	**18-29 Years**	8.7	(7.0-10.6)
Low education	22.0	(16.8-28.4)	Low education	11.5	(7.9-16.5)
Medium education	14.9	(12.6-17.4)	Medium education	7.0	(5.2-9.5)
High education	5.7	(10.1-12.9)	High education	9.6	(6.2-14.8)
**30-44 Years**	11.4	(10.1-12.9)	**30-44 Years**	9.5	(8.0-11.3)
Low education	19.0	(13.6-25.9)	Low education	13.1	(8.5-19.7)
Medium education	9.9	(8.4-11.7)	Medium education	10.4	(8.2-13.2)
High education	10.6	(8.6-12.9)	High education	5.9	(4.5-7.8)
**45-64 Years**	14.4	(13.3-15.7)	**45-64 Years**	15.9	(14.7-17.3)
Low education	18.5	(15.2-22.2)	Low education	19.2	(15.4-23.5)
Medium education	14.1	(12.6-15.8)	Medium education	17.5	(15.6-19.5)
High education	11.8	(10.0-13.8)	High education	11.9	(10.3-13.7)
**≥65 Years**	25.9	(23.9-27.9)	**≥65 Years**	25.8	(23.7-28.0)
Low education	27.9	(24.9-31.1)	Low education	26.5	(22.5-31.0)
Medium education	24.8	(22.1-27.7)	Medium education	26.3	(23.3-29.5)
High education	21.5	(17.2-26.5)	High education	24.7	(21.9-27.7)
**Total (women and men)**	**16.2**	**(15.6-16.7)**	**Total (women and men)**	**16.2**	**(15.6-16.7)**

CI=confidence interval
